# A core outcome set for evaluating self-management interventions in people with comorbid diabetes and severe mental illness: study protocol for a modified Delphi study and systematic review

**DOI:** 10.1186/s13063-017-1805-y

**Published:** 2017-02-14

**Authors:** Johanna Taylor, Jan R. Böhnke, Judy Wright, Ian Kellar, Sarah L. Alderson, Tom Hughes, Richard I. G. Holt, Najma Siddiqi

**Affiliations:** 1 Mental Health and Addiction Research Group, Department of Health Sciences, University of York, York, YO10 5DD UK; 2 Leeds Institute of Health Sciences, University of Leeds, Leeds, LS2 9LJ UK; 3 School of Psychology, University of Leeds, Leeds, LS2 9JT UK; 4 Leeds and York Partnership NHS Foundation Trust, 2150 Century Way, Thorpe Park, Leeds, LS15 8ZB UK; 5 Diabetes and Endocrinology, Human Development and Health Academic Unit, Faculty of Medicine, University of Southampton, Southampton, SO16 6YD UK; 6 University Hospital Southampton NHS Foundation Trust, Tremona Road, Southampton, SO16 6YD UK; 7 Bradford District Care NHS Foundation Trust, New Mill, Victoria Road, Saltaire, Bradford, BD18 3LD UK

**Keywords:** Psychosis, Severe mental illness, Schizophrenia, Bipolar disorder, Diabetes, Self-management, Core outcome set, Comorbidity, Complex interventions

## Abstract

**Background:**

People with diabetes and comorbid severe mental illness (SMI) form a growing population at risk of increased mortality and morbidity compared to those with diabetes or SMI alone. There is increasing interest in interventions that target diabetes in SMI in order to help to improve physical health and reduce the associated health inequalities. However, there is a lack of consensus about which outcomes are important for this comorbid population, with trials differing in their focus on physical and mental health. A core outcome set, which includes outcomes across both conditions that are relevant to patients and other key stakeholders, is needed.

**Methods:**

This study protocol describes methods to develop a core outcome set for use in effectiveness trials of self-management interventions for adults with comorbid type-2 diabetes and SMI. We will use a modified Delphi method to identify, rank, and agree core outcomes. This will comprise a two-round online survey and multistakeholder workshops involving patients and carers, health and social care professionals, health care commissioners, and other experts (e.g. academic researchers and third sector organisations). We will also select appropriate measurement tools for each outcome in the proposed core set and identify gaps in measures, where these exist.

**Discussion:**

The proposed core outcome set will provide clear guidance about what outcomes should be measured, as a minimum, in trials of interventions for people with coexisting type-2 diabetes and SMI, and improve future synthesis of trial evidence in this area. We will also explore the challenges of using online Delphi methods for this hard-to-reach population, and examine differences in opinion about which outcomes matter to diverse stakeholder groups.

**Trial registration:**

COMET registration: http://www.comet-initiative.org/studies/details/911. Registered on 1 July 2016

## Background

Severe mental illness (SMI) is a term used to describe illnesses in which psychosis occurs (e.g. schizophrenia, schizoaffective disorder, and bipolar disorder). Diabetes is two to three times more common in people with SMI compared to the general population [1], and is associated with worse health outcomes [2]. Higher prevalence and poor management of diabetes is thought to contribute significantly to the lower life expectancy of people with SMI [3], which is around 15 years lower than the general population [4, 5]. Poor diabetes management leads to complications including heart disease, stroke, foot ulceration and amputation, eye and kidney disease [6], many of which can be prevented with better diabetes care. In England, it is estimated that approximately 50,000 people with SMI also have diabetes [7], a figure that is expected to grow in line with the growing numbers of people with both diabetes and mental illness; recent evidence suggests that prevalence of diabetes is increasing at a faster rate in people with SMI than in people without [1].

Various pharmacological and behavioural interventions to prevent diabetes or to improve its outcomes have been tested in people with SMI [8–11]. Many of these target metabolic and cardiovascular risk factors, with the majority focusing on the metabolic side effects of antipsychotic medications, such as olanzapine and clozapine, which are commonly prescribed in SMI [10]. Interventions typically include physical activity and weight loss programmes [12, 13]; behaviour change techniques that seek to promote positive lifestyle changes (e.g. problem solving and goal setting) [14, 15]; antidiabetic and weight loss medications [16, 17]; switching to, or adding, an antipsychotic medication associated with fewer metabolic side effects [18, 19]; and other drugs (e.g. hypnotics and antiepileptic medications) [20, 21], which are thought to alter metabolic functioning, anthropological markers, or other important risk factors (e.g. blood pressure, lipid levels).

While few studies have focused specifically on people with coexisting diabetes and SMI [22], several published trials include people with both conditions, and there is a growing interest in developing interventions targeting this comorbidity [23]. Systematic reviews of the evidence consistently find incomplete reporting of outcomes and poor study quality [8–11], due in part to the lack of consensus about what outcomes are important for diabetes comorbid with SMI. This makes it difficult to build an evidence base about what works in this population, with studies differing in their focus on physical and mental health, and their selection of diabetes-related outcomes. For example, in a meta-analysis of lifestyle interventions for people with SMI [11], only eight of 25 included studies measured the effect on glycaemic parameters and only four measured depressive symptoms, despite the fact that diabetes is associated with increased depression and distress [24]. Another systematic review of pharmacological and behavioural interventions reported similar results, with only eight of 33 studies measuring glucose control strategies [9]. This makes it difficult to pool results of different studies and draw conclusions about how to improve diabetes outcomes.

A standardized set of outcomes for interventions targeting comorbid diabetes and SMI is, therefore, required. Core outcome sets, defined as ‘an agreed standardized collection of outcomes … which should be measured and reported in all trials for a specific clinical area’ [25] have already been developed for several conditions and interventions [26]. As well as offering the potential to reduce outcome reporting bias in trials and to enable better aggregation of results across multiple studies, a core outcome set aims to include outcomes that are important to all relevant stakeholders, such as patients, carers, health care professionals, and commissioners [25], and will, therefore, include outcomes beyond purely clinical measurements (e.g. patient-reported outcomes or reports from caregivers).

Previous studies have employed various approaches to determine which outcomes to consider for inclusion in a core outcome set, (e.g. systematic reviews and qualitative inquiry), and to reach consensus about the outcomes that are most important (e.g. ranking methods such as Delphi and Nominal Ranking Technique) [26]. A recent systematic review that aimed to increase understanding about the methods used in the growing number of core outcome set studies found no completed studies relevant to either the SMI or diabetes populations [26]. Five pertinent studies are currently registered on the Core Outcome Measures in Effectiveness Trials (COMET) database [27]. There are two ongoing studies aiming to develop a core outcome set: one for the treatment of type-2 diabetes in adults [28], and the other for people with schizophrenia and bipolar disorder living in the community [29]. Two studies have focused on identifying outcomes already employed in trials. One examined the first 10,000 trials on the Cochrane Schizophrenia Group’s Register and found that 2194 instruments were used to measure outcomes of 1940 different interventions [30]. Another examined whether diabetes trials included outcomes important to people with diabetes such as death and quality of life; 201 of 436 trials included them as a primary or secondary outcome [31]. The final study sought patient preferences about diabetes outcomes. Reducing risk of death was selected as the most important outcome (followed by reducing glycosylated haemoglobin (HbA_1c_)), but preventing kidney failure and need for dialysis was the most frequently endorsed [32].

Other work relevant to identifying important diabetes outcomes includes the American Association of Diabetes Educators’ (AADE) Diabetes Education Outcomes Project, which provides a framework for benchmarking and universal measurement of diabetes self-management education (DSME) interventions [33]. The DSME Outcomes Continuum includes immediate (e.g. knowledge, skills); intermediate (e.g. healthy eating, physical activity); post-intermediate (e.g. HbA_1c_, Body Mass Index (BMI)); and long-term outcomes (e.g. quality of life), which, while particularly pertinent for DSME programmes, are also relevant to self-management of other long-term conditions. Knowledge and skills, improved quality of life, and biomedical markers were also highlighted in a systematic review of stakeholder views of self-management outcomes [34]. The review identified other important outcomes as well, including independence, relationships with health care providers, wellbeing, and managing stress. Additionally, the review highlighted a lack of consensus between people with diabetes, carers, and health professionals.

While these studies help to inform the identification of outcomes that are relevant for people with either SMI or diabetes, simply combining outcomes for diabetes and SMI may not capture what is most important for people living with both conditions together [35]. Self-management is the cornerstone of good diabetes management [36]. However, people with SMI have motivational, cognitive, and psychological deficits that may impact on their ability to manage their diabetes. Outcomes of self-management interventions in this population may, therefore, be different from those without SMI. Additionally, diabetes is associated with an increased risk of depression and distress, which is an especially important consideration for people who already have an underlying mental illness [24]. Coexisting physical and mental illness is likely to affect people in ways that are different from having one condition alone, including the medication they take, and how they interact with health services and manage their health on a daily basis [37]. Relevant outcomes are, therefore, likely to cover multiple health domains, including functional, physical, cognitive, and emotional.

Our group is currently undertaking a programme of research to develop and evaluate a DSME programme tailored for people with coexisting type-2 diabetes and SMI, the ‘Diabetes and Mental Illness – Improving Outcomes and Services (DIAMONDS)’ programme. No DSME intervention has been tested previously in this population, and to date only one published randomised controlled trial has specifically targeted people with both diabetes and SMI [38]. To support the DIAMONDS programme of work, a necessary first step is to develop a core outcome set for DSME interventions targeting people with coexisting type-2 diabetes and SMI. The purpose is to ensure that evidence about what works for this population can be developed and aggregated to offer meaningful conclusions for patients, carers, clinicians, policy-makers, and health care commissioners.

### Aims and objectives

This study aims to develop a core outcome set for self-management interventions targeting adults (age 18 years and over) with coexisting type-2 diabetes and SMI, which captures important domains and is acceptable to people living with both of these conditions and for those who support them.

The study has four objectives, to:

1. *Identify* potential outcomes to consider from existing evidence and through a multistakeholder workshop and service user panel meeting
2. *Rank* outcomes to include in the core outcome set through a two-round online survey with all relevant stakeholders
3. *Agree* which outcomes to include in the set during a multistakeholder consensus workshop
4. *Select* appropriate measurement tools for each outcome in the core outcome set through systematically searching the trials literature, and identify gaps in measures where these exist

## Methods

### Overview

Using a modified Delphi method, this study will employ a three-stage process to (1) Identify, (2) Rank, and (3) Agree core outcomes. The study will comprise a two-round e-Delphi survey and multistakeholder workshops involving patients and carers, clinical professionals, health care commissioners, and other experts (e.g. academic researchers and third sector organisations). In a fourth stage, the study will review existing trials literature to (4) Select appropriate measurement tools for each outcome in the core outcome set and identify gaps where these exist.

This study draws on learning from the OMERACT (Outcome Measures for Rheumatology) collaboration [39]; the COMET Initiative [27]; and a review of methods employed in studies of this type [26], to select appropriate consensus methods for developing the core outcome set, and for ensuring meaningful input from patients and carers. The study will be registered on the COMET website to alert others to our work, and to share learning from our study.

### Step 1: Identify outcomes

1. *Identify* potential outcomes to consider from existing evidence and through a multistakeholder workshop and service user panel meeting

#### Review of existing evidence

We will identify systematic reviews of trials and other experimental studies in people with diabetes and SMI and extract information about outcome measures used in included studies. Only outcomes measured in large studies (more than 20 participants) will be selected, as we seek to identify those practicable to measure in a large trial. We will also incorporate outcomes from the AADE DSME Outcomes Continuum [33] and the systematic review of self-management outcomes that are important to people living with the conditions, carers, and health professionals [34], to form a list of potential outcomes for stakeholders to review.

#### Multistakeholder workshop and service user panel meeting

A multistakeholder workshop will be held, attended by health care professionals and other relevant stakeholders as well as the research group (*n* = 20). Participants will be asked to work in small groups to identify outcomes that are potentially relevant and important. Findings from the review of existing evidence will then be presented, followed by further small group work to identify duplicate and overlapping outcomes, and to develop a final ‘long-list’ of potential outcomes to include in a core outcome set. Following the workshop, the long-list will be presented to a panel of service users with diabetes and SMI and their carers (the DIAMONDS PPI Panel), who will also be given an opportunity to add new outcomes that have not already been identified.

### Step 2: Rank outcomes

2. *Rank* outcomes to include in the core outcome set through a two-round online survey with all relevant stakeholders

An online survey will be administered to determine which outcomes in the list developed in step 1 are important to different stakeholder groups. The purpose of the Delphi study is to reduce the list of potential outcomes to a smaller core set based on the collated responses of participants, but also taking into account the potentially differing views between diverse stakeholder groups and enabling participants to change their views as part of developing a group consensus [40]. Unlike methods like Nominal Ranking Technique, a Delphi study does not require participants to interact to reach a consensus, and instead provides all participants with an equal opportunity to input directly and anonymously. This study draws on methods employed in other relevant studies [29, 41, 42], and also takes into account the potential burden on patient and health and social care professional participants of taking part in a study involving several stages. Although potential participants will be encouraged to take part in the online survey, a paper version will be available to ensure that patients and carers in particular are able to take part.

#### Participants

The following stakeholders will be purposively sampled to ensure good representation across the different groups:

1. Adults with coexisting SMI (which for this study we define as illnesses in which psychosis occurs including schizophrenia, schizoaffective disorder, bipolar disorder and severe depression) and type-2 diabetes living in the community (*n* = 5–10)
2. Carers/supporters (expected to be a spouse, other family member or close friend providing regular care or support to a person with SMI and diabetes) (*n* = 5–10)
3. Health and social care staff (to include GPs, diabetologists, psychiatrists, psychologists, nurses, mental health care co-ordinators, social workers, and other staff supporting this patient group) (*n* = 15–20)
4. Health service managers and commissioners (*n* = 5–10)
5. Academic experts in diabetes, mental illness, primary care and outcome measurement (*n* = 15–25)

Sample sizes vary across Delphi studies; based on previous work [26] and taking into account the small target patient population, this study will aim to recruit between 50 and 75 participants to the first round. To ensure good representation of patients, the DIAMONDS PPI Panel will also be invited to take part in the Delphi study (*n* = 5–10).

#### Recruitment

Patients and carers will be recruited through Community Mental Health Teams (CMHTs) based in participating mental health NHS trusts in one region of England, using care co-ordinators (mental health nurses, occupational therapists, social workers, and support workers who case manage individuals with SMI) to identify and invite eligible patients on their caseloads whom they assess as having capacity to consent. Other participants will be recruited through NHS and third sector organisations involved in the wider research programme. Participants will be informed that there will be two stages in the survey. We will also seek endorsement for this work from professional organisations to assist with recruitment for the Delphi study and also adoption of the core outcome set into research and practice.

#### Delphi round-1 survey

The survey will be designed and administered using Qualtrics [43], a secure web-based survey tool used successfully in research with mental health service users [44] and staff supporting people with SMI [45]. All participants will be assigned a unique identifier and basic demographics will be collected to assist with the analysis and collation of responses. During round 1, all potential outcomes will be presented to participants, who will be asked to rate the importance of each outcome on a scale with anchors ranging from 1, being not important to 9, being of critical importance.

Using free-text boxes participants will have an opportunity to provide written feedback about their choices, and to suggest additional outcomes that they believe are important. Participants will be given 4 weeks to complete the survey – response rates will be monitored throughout to ensure good representation across the participant groups. If a stakeholder group is under-represented in the survey (fewer than five participants), a further attempt to recruit additional participants will be made.

#### Round-1 analysis

Data will be analysed by stakeholder group and for all participants together. For each outcome, the number of respondents and distribution of scores will be summarised and analysed. The proportion of participants scoring 1–3, 4–6, and 7–9 for each outcome will also be calculated. Text data provided by participants in the free text fields will be reviewed by the research team to identify new outcomes to include in round 2 of the Delphi study survey and delete or merge duplicate or overlapping concepts.

#### Delphi round-2 survey

Participants from round 1 will be invited to participate in round 2. As outlined above, we will take precautions to reduce dropout for this second stage, including clearly informing participants of the two stages in the survey. If respondents from the first round cannot participate in the second, we will seek to recruit additional participants from the same stakeholder group if they are under-represented.

Participants will be presented with the following findings from round 1: ranking of outcomes for the whole group, ranking of outcomes for their own stakeholder group, and their individual scores. Participants will then be asked to rate each outcome again using the same Likert scale so that participants can adjust their scores, and to allow for comparisons between round 1 and round 2.

#### Round-2 analysis

Drawing on consensus methods used in similar studies [26], data will be analysed by stakeholder group and for the whole group to determine for each outcome, the percentage of respondents who have scored 1–3, 4–6, and 7–9.

Each outcome will be categorised as one of the following:

- ‘*Consensus In*’ (i.e. the outcome should be included in the core outcome set): more than 70% of participants score the outcome as 7–9 (important) *and* fewer than 25% score the outcome as 1–3 (not important)
- ‘*Consensus Out*’ (i.e. the outcome should not be included in the core outcome set): more than 70% score the outcome as 1–3 (not important) *and* fewer than 15% score the outcome as 7–9 (important)
- ‘*No Consensus*’ (i.e. there is no strong consensus about the importance of the outcome): any other distribution of scores

We will analyse in detail the consistency of these results within groups as well as across groups to make statements about the relevance of the outcomes across all participating stakeholders, and to ensure that no voices remain unheard and that minority stakeholders are not over-ruled by the majority.

### Step 3: Agree outcomes

3. *Agree* which outcomes to include in the core outcome set during a multistakeholder consensus workshop

#### Delphi consensus workshop

The detailed results from the survey will be presented at a Delphi workshop to be attended by people with SMI and type-2 diabetes, carers, health and social care professionals, health care commissioners, and other relevant stakeholders as well as the research group (*n* = 20). We anticipate that patients may prioritise outcomes in the Delphi study that practitioners or commissioners may not, and vice versa. It is, therefore, important that we include the views of all relevant stakeholders in this workshop to reach a decision about outcomes for which there was no clear consensus in the Delphi study. To ensure that we have meaningful input across participant groups, people participating in the Delphi study will be invited to attend the workshop, and for groups that are difficult to recruit for this part of the study we will make efforts to seek their views in advance of the workshop. We will also consult with the DIAMONDS PPI Panel separately prior to the workshop so that their views about the results of the Delphi study can be incorporated into the final selection process.

The purpose of the consensus workshop is to agree a final set of outcomes for the core outcome set, using the detailed findings from the survey. This is a key element of the modified Delphi method which combines self-administered questionnaires and physical meetings [46]. All outcomes will be discussed. Each ‘Consensus In’ outcome will be discussed to ensure that it is measurable and feasible to include in the core set. Each ‘Consensus Out’ and ‘No Consensus’ outcome will be assessed to ensure that key outcomes that are commonly used to make policy or commissioning decisions about adoption have not been excluded from the set (e.g. outcomes used for cost-effectiveness analyses). The detailed results for each ‘No Consensus’ outcome will be discussed to determine whether they should be included in the final set. To ensure that there is no duplication in the final proposed set, each outcome will be discussed to ensure that it relates to a distinct construct and to start identifying existing measurement tools (and key validation studies of measurement tools) in preparation for step 4.

Finally, workshop participants will be asked to select the two outcomes that they consider most important to assess for diabetes and SMI populations from the proposed core outcome set.

### Step 4: Identify instruments to measure outcomes

4. *Select* appropriate measurement tools for each outcome in the core outcome set, and identify gaps in measures where these exist

We will adopt a pragmatic approach to identify measurement tools as follows:

1. Discussion during the final consensus workshop to identify commonly used tools that are well validated (e.g. some outcomes will be measured consistently with one tool which has been validated in a key paper). Key papers for identified outcomes will be assessed against the COnsensus-based Standards for the selection of health Measurement INstruments (COSMIN) Checklist [47] which uses a rating scale to assess the quality of measurement properties
2. For the remaining outcomes, a systematic review of published studies on the properties of all available measurement instruments that aim to measure the particular construct will be conducted. Study criteria and methods for the systematic review will be adapted depending on the final core outcome set. A search strategy comprising text words and index terms will be developed using a search filter for outcome measure properties [48] and search terms for each of the outcomes in the final core outcome set. Results identified from the search will be screened by two researchers independently, first by title and abstract to remove irrelevant studies, and second by full text to identify all papers that provide details of potentially matching measurement tools. The measurement and psychometric properties of all potentially matching tools will be assessed using the COSMIN Checklist [47] before selecting which tools to include for outcomes in the core outcome set

Outcomes for which no matching validated measurement tool is identified will be highlighted in the review to inform future research priorities.

## Discussion

To ensure that evidence about what works for particular populations can inform policy and practice and improve the quality and effectiveness of health care, evaluation research must measure what matters to people living with the condition and those who support them and commission health services, as well as to researchers. Developing core outcome sets using appropriately adapted consensus and trials literature review methods facilitates this, and helps to increase consistency of measurement in future research thereby enhancing the potential to combine trials for evidence synthesis. The proposed core outcome set for this study will provide clear guidance about what outcomes should be measured, as a minimum, in trials of self-management interventions for people with coexisting type-2 diabetes and SMI, and help to improve future aggregation of trial evidence in this area.

There are several challenges that we are likely to encounter. First, the study risks not including representation across stakeholder groups, in particular patients and carers. People with SMI are a hard-to-reach group; however, working with mental health care co-ordinators to identify and recruit participants is an approach used successfully in other research [49]. People with SMI are also less likely than the general population to have regular access to the Internet [50], which is a prerequisite for completing an online survey. The Delphi study will therefore, as needed, be administered by post, telephone or in person, as well as online. To promote continued engagement we will endeavour to make sure that the survey is easy to complete by piloting this with the DIAMONDS PPI Panel, and we will recruit additional participants if we experience dropout in this or other stakeholder groups. There is a risk that during the consensus workshop, the voice of patients and carers is not present or is under-represented. The DIAMONDS PPI Panel members have expressed their desire to meet separately and to have their views presented at the workshop instead of attending in person. To ensure that other patient and carer participants are able to contribute to this process, we will use a skilled facilitator during the workshop and also provide them with an opportunity to meet separately with a researcher if they prefer.

Second, we may find differences in opinion between diverse stakeholder groups as they seek to achieve consensus about which outcomes are important. Core outcome sets are a relatively new construct in research, and methods for reaching consensus are evolving as studies begin to reflect on their successes and limitations [25]. A key feature of the Delphi survey is building consensus by including more than one round, and sharing the results of each round with participants, so that these can inform the choices that individuals subsequently make. However, there are likely to be multiple outcomes that do not reach consensus, and the modified Delphi method provides an opportunity to resolve areas of disagreement between different stakeholder groups and ensure that all groups are represented in this process. There are examples in the trials literature where outcomes that are discarded by consensus are included in a core outcome set or vice versa [51]. While this may be contentious, a flexible and pragmatic approach allows for decisions to be taken which are based on the consensus opinion but also take account of the necessity to include measurable and appropriate outcomes.

A flexible approach that acknowledges the expert in the final selection process also helps to address a third challenge, which all research of this type will face in the context of rapidly developing medical technologies and expanding psychometric and epidemiological evidence: the accuracy and stability of the concepts guiding our research (SMI and diabetes). For example, current research into the nature of SMI questions the meaningfulness of existing diagnostic categories, both aetiologically [52, 53] and phenomenologically [54]. Thus, the appropriateness of grouping together SMI conditions that display similar and overlapping genetic aetiology and symptomatology, but have different illness trajectories and treatments, continues to be debated [53]. In diabetes, although supporting self-management has been the cornerstone of good diabetes care for many years, it is a multidimensional concept that is individualised by those living with diabetes and, therefore, challenging to measure reliably [55, 56]. Concerns about increasing prevalence of diabetes and associated costs are fuelling the development of new treatments and therapies, which introduces a slightly different set of questions about the management of diabetes and the potential outcomes that capture this.

A fourth challenge relates to patient-reported outcomes, which are commonly used to evaluate the effectiveness of interventions. Psychometric assessments of these have shown that such measures overlap in content [57], and the incremental value of using multiple measures might, therefore, be questionable [58]. While the development of these measures is informed and partly motivated by their value as an accepted outcome criterion in care and trials [59], the pressure on researchers to provide evidence on the mechanisms that drive change in an intervention has risen [60]. This complex landscape necessitates detailed reviews of outcome assessment strategies as well as sophisticated strategies to define informative, relevant and sufficiently independent sets of outcome criteria. To assess this, potential outcome measures can be categorised according to their functional relationship in an intervention, in other words to differentiate whether an outcome measure could serve as an immediate outcome of the intervention itself (e.g., content learned); whether it is a mediator that is meant to change a primary or distal outcome (e.g., self-management skills); or whether it could serve as a primary outcome in a trial (e.g., measures of glycaemic control and/or psychological distress). This exercise could also draw on existing frameworks such as the DSME Outcomes Continuum [33] and standardised approaches for the evaluation of interventions [61].

Finally, there are challenges to ensuring that the core outcome set we develop is feasible to implement and accessible to those involved in research in this area. It is quite possible that the core outcome set will include outcomes for which we identify no appropriate measurement tool, or one that has not been adequately validated. If this occurs, we will seek funding to carry out this work or work in collaboration with others to validate tools that already exist. Historically, research has been poor at disseminating to different audiences and translating evidence into practice [62]. To ensure that the core outcome set we develop is accessible, we will update the COMET website in a timely manner, and make sure that the core outcome set is publicly available and disseminated through our mental health and diabetes research and practice networks.

### Study status

At the time of manuscript submission the status of the core outcome set study is ongoing. Participant recruitment for the Delphi study has not yet commenced.
